# Microenvironmental Modulation of Decorin and Lumican in Temozolomide-Resistant Glioblastoma and Neuroblastoma Cancer Stem-Like Cells

**DOI:** 10.1371/journal.pone.0134111

**Published:** 2015-07-31

**Authors:** Cristiano Farace, Jaime Antonio Oliver, Consolacion Melguizo, Pablo Alvarez, Pasquale Bandiera, Ana Rosa Rama, Giulia Malaguarnera, Raul Ortiz, Roberto Madeddu, Jose Prados

**Affiliations:** 1 Department of Biomedical Sciences, University of Sassari, Sassari, Italy; 2 Institute of Biopathology and Regenerative Medicine (IBIMER), Granada, Spain; 3 Department of Anatomy and Embryology, University of Granada, Granada, Spain; 4 Biosanitary Institute of Granada (ibs.Granada), SAS-University of Granada, Granada, Spain; 5 Department of Health Science, University of Jaén, Jaén, Spain; 6 Research Center "The Great Senescence", University of Catania, Catania, Italy; 7 National Institute of Biostructures and Biosystem (INBB), Rome, Italy; University of Crete, GREECE

## Abstract

The presence of cancer stem cells (CSCs) or tumor-initiating cells can lead to cancer recurrence in a permissive cell–microenvironment interplay, promoting invasion in glioblastoma (GBM) and neuroblastoma (NB). Extracellular matrix (ECM) small leucine-rich proteoglycans (SLRPs) play multiple roles in tissue homeostasis by remodeling the extracellular matrix (ECM) components and modulating intracellular signaling pathways. Due to their pan-inhibitory properties against receptor tyrosine kinases (RTKs), SLRPs are reported to exert anticancer effects *in vitro* and *in vivo*. However, their roles seem to be tissue-specific and they are also involved in cancer cell migration and drug resistance, paving the way to complex different scenarios. The aim of this study was to determine whether the SLRPs decorin (DCN) and lumican (LUM) are recruited in cell plasticity and microenvironmental adaptation of differentiated cancer cells induced towards stem-like phenotype. Floating neurospheres were generated by applying CSC enrichment medium (neural stem cell serum-free medium, NSC SFM) to the established SF-268 and SK-N-SH cancer cell lines, cellular models of GBM and NB, respectively. In both models, the time-dependent synergistic activation of DCN and LUM was observed. The highest DCN and LUM mRNA/protein expression was detected after cell exposure to NSC SFM for 8/12 days, considering these cells as SLRP-expressing (SLRP^+^) CSC-like. Ultrastructural imaging showed the cellular heterogeneity of both the GBM and NB neurospheres and identified the inner living cells. Parental cell lines of both GBM and NB grew only in soft agar + NSC SFM, whereas the secondary neurospheres (originated from SLRP^+^ t_8_ CSC-like) showed lower proliferation rates than primary neurospheres. Interestingly, the SLRP^+^ CSC-like from the GBM and NB neurospheres were resistant to temozolomide (TMZ) at concentrations >750 μM. Our results suggest that GBM and NB CSC-like promote the activation of huge quantities of SLRP in response to CSC enrichment, simultaneously acquiring TMZ resistance, cellular heterogeneity, and a quiescent phenotype, suggesting a novel pivotal role for SLRP in drug resistance and cell plasticity of CSC-like, allowing cell survival and ECM/niche modulation potential.

## Background

Glioblastoma (GBM) and neuroblastoma (NB) are the most common and lethal nervous system malignant cancers in adult and pediatric patients, respectively. The World Health Organization considers GBM the most aggressive astrocytoma, and it can develop into secondary GBM from low-grade gliomas, or *de novo* with rapid progression to death [1]. In contrast, NB mainly arises from neural crest cells as a neuroendocrine cancer of the sympathetic nervous system, and 60% of pediatric patients show metastatic disease at diagnosis [2]. Although temozolomide (TMZ)–an alkylating agent which induces cell death by whole DNA alkylation/methylation in guanine residues–in combination with other drugs or radiotherapy represent a first-line treatment increasing the overall survival (OS) of patients with GBM or NB [3, 4], drug resistance and cancer progression are common. Because GBM is highly invasive in the brain and NB tends to invade other organs, patient OS remains poor (< 1.5 years in GBM patients and 4 years in NB patients) [5, 6].

Treatment failure in cancer patients has previously been related to cancer stem cell (CSC) subpopulations, which ensure the maintenance of cancer heterogeneity, and these CSC subpopulations are more resistant to selective drugs through multiple concerted steps of self-renewal and differentiation [7–9]. Metastasis and cancer recurrence are also linked to the behavior of CSCs, including their quiescent phenotype, migratory ability, and evasion of the immune system [10]. Abundant research suggests that cells stem-like cells are equipped with innate machinery that protects them from radio/chemotherapy [11, 12]. This includes stem-related mechanisms, such as protective cell niches and changes in the expression of genes involved in the regulation of the cell cycle, DNA repair, drug metabolism, and drug efflux [13]. The drug resistance and cellular invasion potential of CSCs also increase at the reversible epithelial-to-mesenchymal phenotypic transition (EMT) [14, 15], which recapitulates the EMT in normal organogenesis and development [16, 17].

Several microenvironmental signals, including the reorganization of the extracellular matrix (ECM), hypoxia, and autocrine/paracrine factors, can determine stem and cancer cell fates [18–25], and trigger or inhibit EMT processes [26, 27]. Therefore, ECM glycoproteins and proteoglycans that are capable of modifying both the ECM environment and intracellular signaling pathways are of utmost importance in the cancer microenvironment [28–30]. The small leucine-rich proteoglycans (SLRPs), sharing strategically conserved domains, represent a clear example of the abovementioned concept. The leucine-rich protein core (40–50 kDa) bind to a number of growth factors (GF) and membrane receptors, whereas ramification of glycosaminoglycanic side chains are involved in ECM–collagen assembly and also in membrane receptor binding. Interestingly, in spite of their pan-inhibitory properties against receptor tyrosine kinases (RTKs) and cancer growth pathways, the “guardian from the matrix” decorin (DCN) and lumican (LUM) SLRPs could exert anticancer effects *in vivo* and *in vitro* [31–33]. However, recent studies have shed light on newly identified tissue-specific properties of both DCN and LUM in normal tissues and in the malignant cancer microenvironment. As reported by other authors, the partial glioma inhibition by DCN in gene therapy experiments in rats brings with it a marked reduction of microglial cells infiltration [34], which could affects cancer inhibition *in vivo*. DCN also enhances the evasion of the immune system and muscle invasion in prostate cancer *in vivo* [35], and exerts unexpected protective and antiapoptotic effects in glioma cell lines under hypoxic conditions [36]. In oral malignant squamous cell carcinoma cells, the nuclear localization of DCN seems to enhance cellular invasion *via* the nuclear epidermal growth factor receptor (EGFR) pathway [37, 38], whereas in osteosarcoma cells, DCN-mediated growth arrest is avoided *via* the protracted activation of membrane EGFR [39]. Clinically, DCN has been proposed as regulator of chemoresistant mechanism in oral cancer [40] and related to drug resistance and reduced survival in GBM patients [41]. Similarly to DCN, LUM is reported to mediate tumor suppression. However, LUM is expressed in high-grade pancreatic cancers with a low degree of differentiation [42] and in GBM patients, as well. LUM also inhibits cell adhesion and promotes the migration of osteosarcoma cells by regulating the transforming growth factor β2 (TGF-β2)/SMAD2 pathway [43], and a 70-kDa LUM proteoglycan seems to enhance cancer cell proliferation and inhibits the migration of pancreatic cancer cells. Moreover, together to DCN, LUM was upregulated in cisplatin-resistant head and neck cancer cells [44].

It is noteworthy that SLRPs are expressed in stem cell niches in the chick embryo [45], in cerebral endothelial cells [46], in progenitors of various cell types [47], and in a NB cell subpopulation unresponsive to nerve-growth-factor-mediated neurite growth [48]. DCN derived from astrocytes also inhibits neural stem cell/progenitor cell differentiation towards a neuron-like cell structure [49]. Altering the mechanical characteristics of three-dimensional (3D) collagen matrices, SLRPs are recruited during the ontogenic (developmental) EMT [50], cell precursor migration and differentiation [51], and wound healing/tissue repair in response to central nervous system injury and inflammation [52]. In this context, it is conceivable that the small DCN and LUM proteoglycans play a role in the biology of CSCs of nervous system origin. To this end, we investigated the involvement of DCN and LUM in GBM and NB CSC-like models, simulating the phenomena of anchorage loss and the detachment of differentiated tumor cells that underlie the EMT process, and their relationship to CSC-like behavior and the cell response to TMZ. In this study, we report for the first time the massive synergistic expression of DCN and LUM SLRPs in GBM and NB cell lines subjected to floating 3D neurosphere-based CSC-like enrichment. Neurosphere micrographs highlight the stem-like heterogeneity and cell polarization of the 3D NB and GBM models. Moreover, SLRP^+^ NB and GBM CSC-like isolated from the neurospheres showed lower proliferation rates, less apoptosis, and greater drug resistance than the parental cell lines, suggesting pivotal and synergistic roles for DCN and LUM in the TMZ resistance, survival, and maintenance of quiescent, slow-cycling, CSC-like subpopulations.

## Methods

### Cell lines and CSC enrichment

In this study, two established GBM and NB cell lines were enrolled. The SK-N-SH cell line is a commercial epithelial cell line originally derived from bone marrow metastasis of a 4-years-old Caucasian female suffering with NB, and it was previously enriched in CSC-like. In contrast, SF-268 is a nonepithelial cell line derived from a high-grade anaplastic astrocytoma, which has never been used for CSC-like enrichment. The established SK-N-SH (from the American Type Culture Collection) and SF-268 cancer cell lines (kindly provided by the Instrumentation Scientific Center, Granada University) were routinely maintained as adherent cultures (monolayers) in Dulbecco’s modified Eagle’s medium (DMEM) supplemented with 10% fetal bovine serum and 1% penicillin/streptomycin, in a humidified atmosphere at 37°C with 5% CO_2_. Confluent cells were detached in 5 ml of phosphate-buffered saline (PBS)–ethylenediaminetetraacetic acid (EDTA) at 37°C for 10 min, washed twice in PBS, and subcultured as monolayers. CSC enrichment of the cancer cell lines was performed by generating neurospheres in neural stem cell serum-free medium (NSC SFM) [53, 54], containing KnockOut DMEM/F12 plus 20 μg/ml basic fibroblast growth factor (bFGF), 20 μg/ml epidermal growth factor, 1× StemPro Neural Supplement (Invitrogen, Paisley, UK), 1% l-glutamine, and 1% penicillin/streptomycin. NSC SFM was replaced every 2 days after mild centrifugation of the neurospheres.

### RNA extraction and quantitative reverse transcription (qRT)–PCR

The expression of DCN and LUM mRNAs was assessed in t_0_ cells and neurospheres after 4 (t_4_), 8 (t_8_), and 12 days (t_12_) of CSC enrichment. The parental cell lines in DMEM were used as the control (t_0_). RNA was extracted with the RNeasy Mini Kit (Qiagen, MD, USA) and quantified with a Nanodrop spectrophotometer (Thermo Scientific, DE, USA). The RNA (1 μg) from each sample was reversed transcribed with M-MLV reverse transcriptase (Sigma, Italy), according to manufacturer’s instructions, and SYBR Green-based amplification (Applied Biosystems, Foster City, CA) was performed with the CFX96 Real-Time PCR Detection System (Bio-Rad, Italy), as previously reported [55]. The PCR cycling program was: 50°C (2 min), 95°C (2 min), 42 cycles of: denaturation at 95°C (30 s), annealing at 56°C (30 s), and extension at 72°C (40 s), followed by a melting curve analysis (range 56–95°C) with increments of 0.5°C/5 s to assess the primer specificity. The primer sequences were: forward 5′-GGA CCG TTT CAA CAG AGA GG-3′, reverse 5′-GAC CAC TCG AAG ATG GCA TT-3′ (DCN); forward 5′-TGG AGG TCA ATC AAC TTG AGA A-3′, reverse 5′-CAA ACG CAA ATG CTT GAT CTT-3′ (LUM); forward 5′-CAA GGA GTA AGA CCC CTG GAC-3′, reverse 5′-TCT ACA TGG CAA CTG TGA GGA G-3′ (glyceraldehyde 3-phosphate dehydrogenase, GAPDH); forward 5′-GGC ATC CTC ACC CTG AAT GA-3′, reverse 5′-AGG TGT GGT GCC AGA TTT TC-3′ (β-actin, ACTB). The target transcripts were independently normalized to GAPDH and ACTB (housekeeping genes), and the RNA of the t_0_ cells was used as the calibration control. The results were expressed on a logarithmic scale as fold changes (FCs), with the 2^–ΔΔCt^ method.

### Western blotting

Whole protein extracts were obtained with the pulsed sonication of cellular pellets in 200 μl of extraction buffer containing 50 mM Trizma-base, 0,25 mM sucrose, 5 mM EDTA (pH 7.4), 0.5% Triton-X 100 and 1% protease inhibitor cocktail. The protein concentrations were determined with the Bradford method. The proteins (50 μg) were mixed 1:1 with 2 × Laemmli buffer, heated at 95°C for 5 min, and loaded onto a 12% denaturing SDS-PAGE gel with the Kaleidoscope prestained standards. The proteins were separated electrophoretically at a constant voltage (90 V) for 90 min and blotted onto a 0.45 μm nitrocellulose membrane under semidry conditions with the Trans-Blot SD Semi-Dry Electrophoretic Transfer Cell (Bio-Rad, Spain) for 25 min at 200 mA. The membranes were blocked with 5% skim milk in PBS, incubated at 4°C overnight with rabbit polyclonal anti-LUM antibody (diluted 1:100; sc-33785, Santa Cruz Biotechnology, Santa Cruz, CA) or mouse monoclonal anti-DCN antibody (1:50; sc-73896, Santa Cruz Biotechnology) in blocking buffer, washed 4 times in washing solution (0.1% Tween in PBS), incubated at room temperature for 1 h with the appropriate monoclonal horseradish-peroxidase-conjugated secondary antibody (1:5000; Sigma Aldrich), and washed again in washing solution. The blots were detected with ECL Western Blotting Detection Reagents (Amersham; UK). The Molecular Imager VersaDoc MP 4000 system (Bio-Rad, Hercules, CA) was used for chemiluminescence visualization. The blots were stripped and incubated with mouse monoclonal anti-β-actin antibody (1:30000; Sigma Aldrich) as the loading control.

### Soft agar cultures

The cell lines were cultured in soft agar to assess their colony or neurosphere formation under anchorage-independent conditions, in DMEM or NSC SFM. To explore the proliferation of the SLRP^+^ CSC-like after CSC enrichment for 8 days (t_8_ CSC-like), secondary neurospheres were generated from t_8_ CSC-like. Briefly, StemPro Accutase Cell Dissociation Reagent (Invitrogen) was used to dissociate the t_8_ neurospheres. A Trypan blue exclusion test was performed and 5 × 10^3^/ml cells were collected in 2 × DMEM or NSC SFM. The cells were mixed 1:1 with a prewarmed solution containing 0.6% ultrapure agarose in PBS (0.3% final agar concentration), seeded in triplicate in six-well plates on 2 ml of solidified 0.6% bottom agar, and incubated in a humidified atmosphere at 37°C with 5% CO_2_. The soft agar cultures were photographed daily under an inverted phase-contrast microscope (Nikon Eclipse TE 2000-S).

### Transmission and scanning electron microscopic imaging

Eight-day (t_8_) neurospheres were collected, washed in PBS, and fixed in 2.5% glutaraldehyde in 0.1 M PBS (pH 7.4) for 2 h at 4°C. The fixed neurospheres were carefully washed four times in PBS, postfixed in 1% osmium tetroxide (OsO_4_) in 0.1 M PBS for 1 h at 4°C, and stored in PBS at 4°C until embedding. The samples used for transmission electron microscopy (TEM) were dehydrated in series of increasing acetone concentrations and embedded in epoxy resin for ultrathin sectioning at 60°C overnight. The ultrathin slices cut with an 8800 Ultratome (LKB, Bromma, Sweden) were stained with 4% uranyl acetate and lead citrate and viewed on a Zeiss EM 109–902 transmission electron microscope (Zeiss, Oberchochen Germany). For scanning electron microscopy (SEM), the postfixed neurospheres were incubated in pure hexamethyldisilazane for 1 h at 4°C, dried in a critical point dryer (Polaron, Watford, UK), and metalized in an S150A Sputter Coater (Edwards, Crawley, UK) for scanning in Quanta 200 (FEI, Eindhoven, The Netherlands) or DSM 962 SEM instruments (Zeiss, Oberchochen, Germany).

### TMZ treatment and MTT assay

Single cells from the monolayers and t_8_ neurospheres of both cell lines were collected as described above. Cell viability was tested with Trypan blue exclusion and 5 × 10^3^ cells/well were seeded in 96-well microtiter plates, in DMEM or NSC SFM. The next day, the medium was replaced with fresh medium without or with different concentrations of TMZ (25–1500 μg/ml; Sigma, Madrid, Spain). DMSO was included as the vehicle control. Each condition was tested in six wells. Three days later, the medium was replaced with fresh medium to preserve TMZ activity. The end-point of the TMZ treatment was established on day 6. Finally, a 3-(4,5-dimethylthiazol-2-yl)-2,5-diphenyltetrazolium bromide (MTT) colorimetric assay was performed (Sigma). Briefly, 20 μl of MTT/well was added and the plates were incubated at 37°C. After 4 h, the medium was carefully removed and DMSO was added to dissolve the formazan salts. The reactions were measured with a Multiskan EX microplate photometer (Thermo Scientific, Madrid, Spain) at 570 nm. The TMZ dose–response was expressed as the percentage (%) inhibition of the cell metabolic activity relative to that of untreated cells and adjusted to the vehicle control. The inhibition of cell growth by TMZ was evaluated in quadruplicate experiments.

### Statistical analysis

Student’s two-tailed *t* test was used to determine the statistical significance of the differences in the TMZ treatment results for the SLRP^+^ CSC-like and the parental cell lines. The differences in qPCR gene expression were evaluated with one-way analysis of variance (ANOVA). Statistical significance was set at p<0.05.

## Results

### DCN and LUM expression during neurosphere-based CSC enrichment

**Fig 1:**
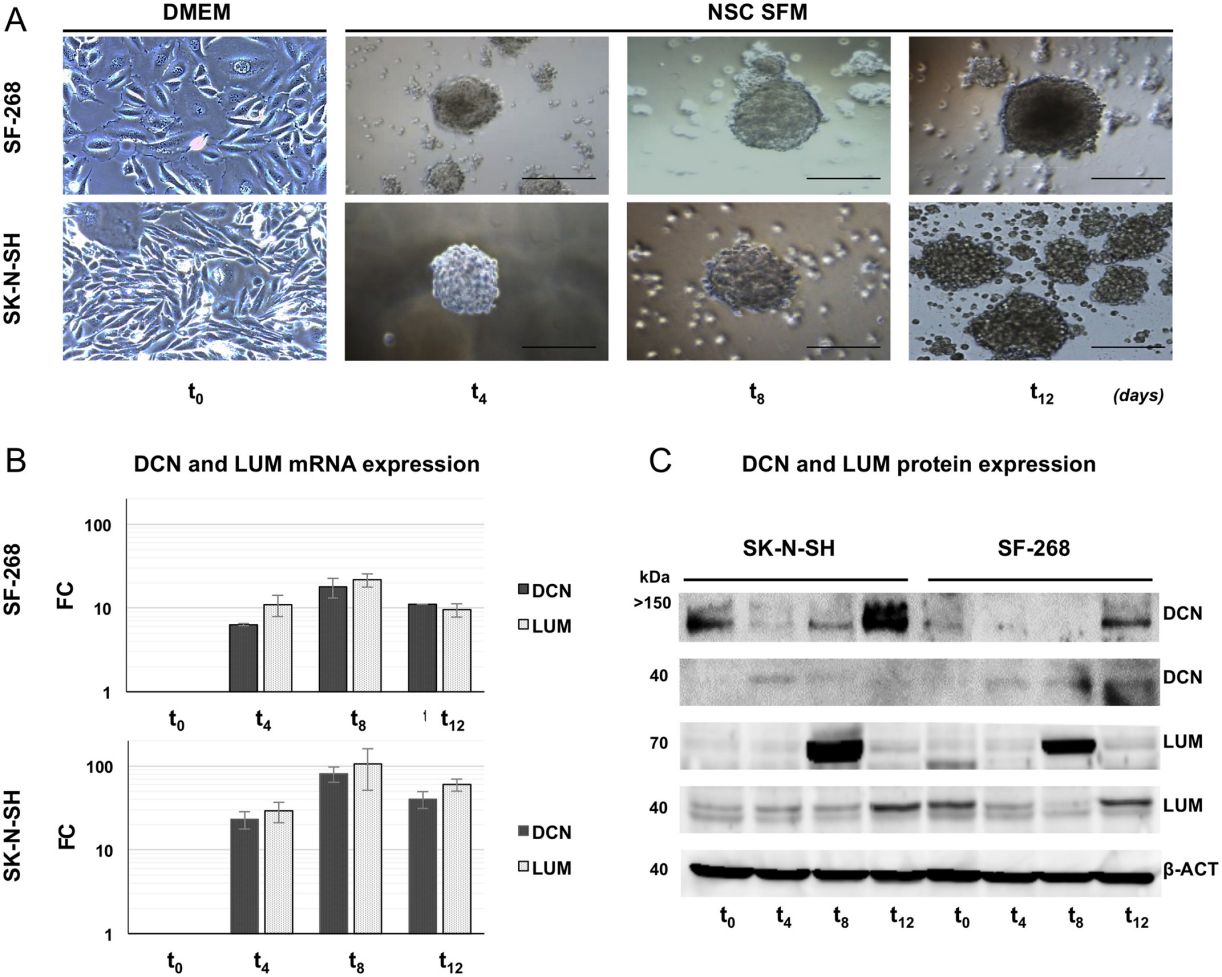
DCN and LUM mRNA/protein expression in GBM and NB CSC-like enrichments. (A) Neurosphere generation from SF-268 and SK-N-SH cell lines. Bar, 50 μm. (B) Expression of DCN and LUM mRNAs in CSC enrichment cultures. qRT–PCR results were normalized to ACTB and graphed as relative fold changes (FCs) between neurospheres at different time points (t4, t8, and t12) and adherent t_0_ cells with the 2^–ΔΔCt^ method. All DCN and LUM FC values for the neurospheres were statistically significant (*p < 0.01). (C) Western blotting analysis of DCN and LUM. β-Actin was used as the loading control. Total DCN and LUM levels were higher in the neurospheres than in the adherent cells of both cell lines. A 70-kDa LUM isoform was upregulated in the neurospheres of both cell lines, with the highest expression in the t8 neurospheres. Adherent SF-268 cells showed basal expression of the 37-kDa LUM core protein.

Neurospheres of both SF-268 and SK-N-SH cell lines acquired regular 3D conformations resulting from the sustained radial proliferation of most cells, reaching >50 μm after 4 days (Fig 1A). A qRT–PCR analysis showed that DCN and LUM mRNA expression, with the exception of LUM in SF-268 t_12_, increased under CSC enrichment conditions in both cell lines, showing the highest DCN and LUM mRNA expression values after 8 days (t_8_). Compared with the parental cell lines (t_0_), and considering ACTB as housekeeping gene, GBM CSC-like were more enriched in LUM mRNA (FC_*Lum*_ = t_0_: 1; t_4_: 11.00; t_8_: 21.70; t_12_: 9.51) than in DCN mRNA (FC_*Dcn*_ = t_0_: 1; t_4_: 6.32; t_8_: 17.87; t_12_: 11.08) (p < 0.01). Similarly, the NB CSC-like showed higher LUM mRNA levels (FC_*Lum*_ = t_0_: 1; t_4_: 29.04; t_8_: 105.78; t_12_: 60.12) than DCN mRNA levels (FC_*Dcn*_ = t_0_: 1; t_4_: 23.02; t_8_: 80.44; t_12_: 40.22) (p < 0.01; Fig 1B).

The protein analysis showed a clear increase in LUM protein (70-kDa) during CSC enrichment. The highest LUM expression was detected in both SF-268 and SK-N-SH t_8_ neurospheres (Fig 1C). By contrast, 40-kDa LUM protein was detected with much less expression that 70-kDa LUM in both SF-268 and SK-N-SH, and especially in t_12_ (Fig 1C). On the other hand, we detected a significant increase in DCN (>150-kDa) protein expression in both SF-268 and SK-N-SH t_12_ neurospheres while 40-kDa DCN protein was detected very weakly, with a difficult expression evaluation. According to mRNA results, 8-days SLRP^+^ CSC-like (t8 CSC-like) derived from both GBM and NB cell lines were enrolled in further analysis.

### Soft agar cultures of SLRP^+^ CSC-like and parental cell lines

**Fig 2:**
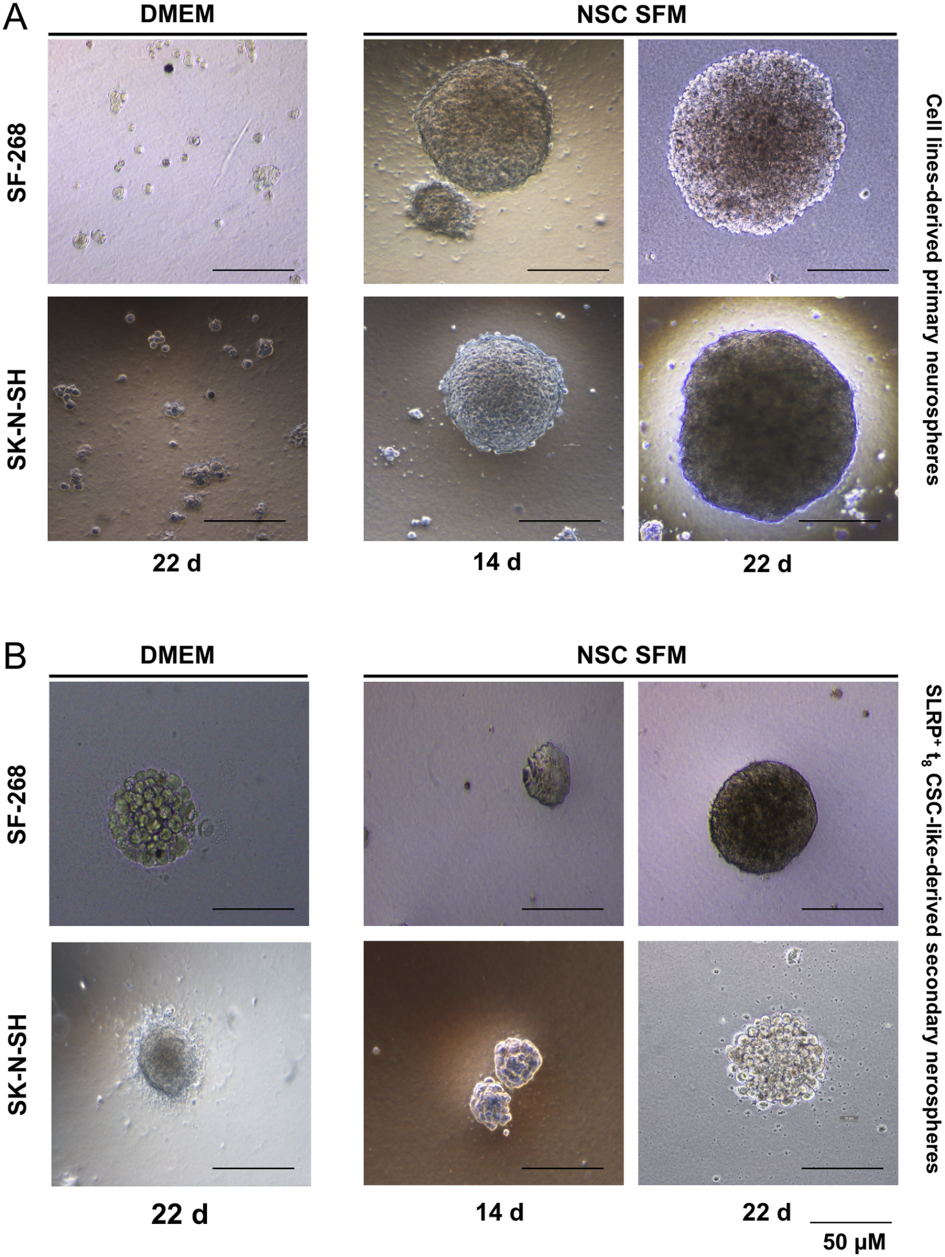
Soft agar cultures of SLRP^+^ CSCs and parental cancer cell lines. (A) Primary soft agar neurospheres from the parental cell lines. Absence of colonies formation in semisolid DMEM (22 days) and neurosphere formation in semisolid NSC SFM (14 and 22 days). (B) Secondary soft agar neurospheres from SLRP^+^ CSC-like isolated from t_8_ neurospheres. Neurospheres were generated in both semisolid DMEM and semisolid NSC SFM (14 and 22 days), suggesting the slow cycling behavior of the CSC-like and residual death evasion activity in the DMEM-grown secondary neurospheres. Bar, 50 μm.

To assess the differences in cell growth between the CSC-like and parental cell lines, the secondary neurospheres from SLRP^+^ t_8_ CSC-like were generated in soft agar and compared with the parental-cell-derived primary neurospheres. The anchorage-independent soft agar cultures showed neurosphere colonies in NSC SFM, whereas no or low colonies of parental cells had grown in the DMEM-based soft agar assay after 22 days (Fig 2A). The primary SK-N-SH neurospheres were larger than the SF-268 neurospheres, reaching > 200 μm after 3 weeks, and had shown a clearly visible dark core within the 3D structure. The soft agar cultures of t_8_ GBM and NB CSC-like (SLRP^+^) grew as small secondary neurospheres in NSC SFM, and curiously also as small colonies in DMEM after 30 days (Fig 2B). The secondary neurospheres were smaller than the primary neurospheres and intrasphere stressed cells (dark cores) were only detectable in the secondary SF-268 neurospheres after 2 weeks, whereas the secondary SK-N-SH neurospheres maintained a translucent appearance.

### Heterogeneic ultrastructures of GBM and NB neurospheres

**Fig 3:**
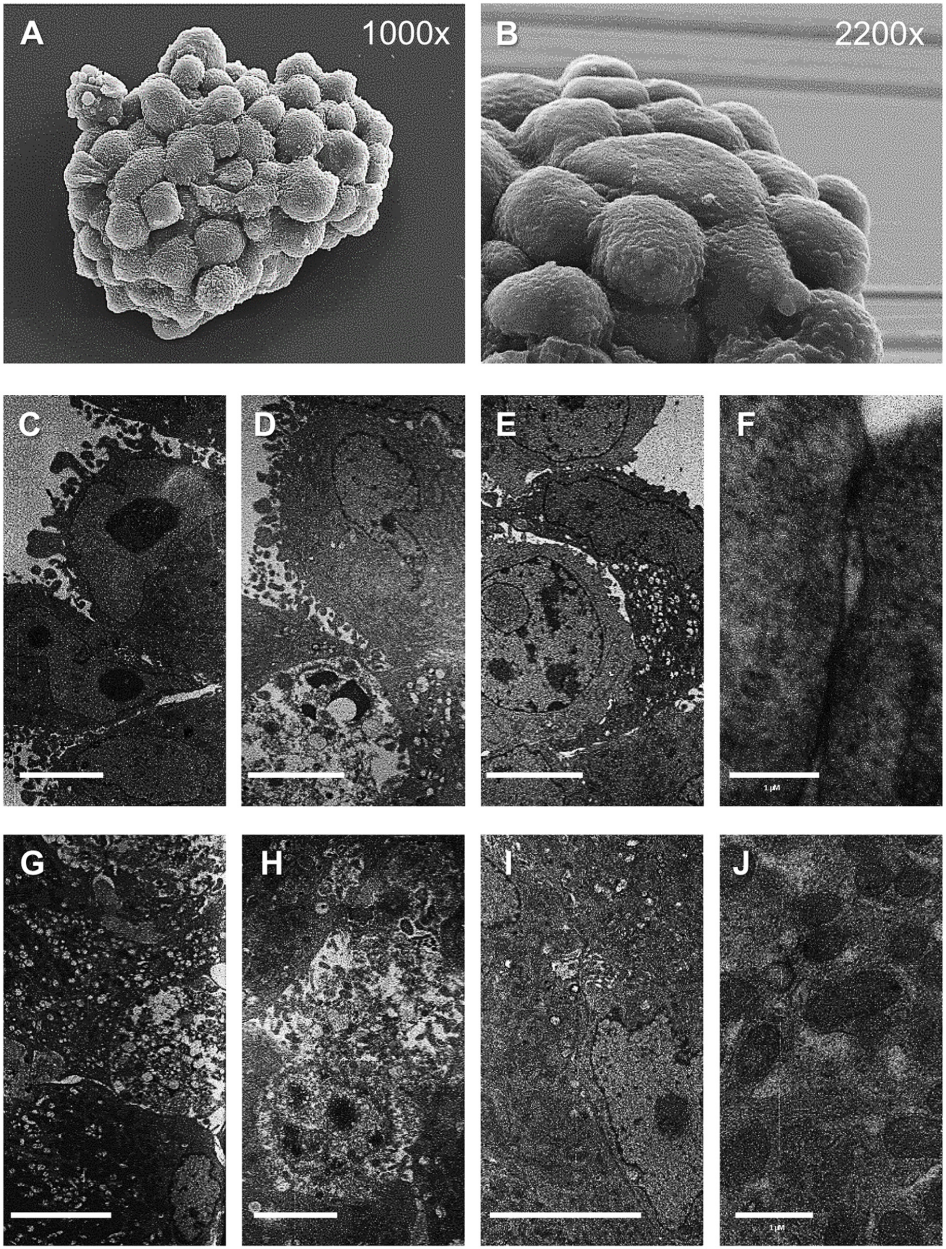
Representative images of GBM t_8_ neurospheres. (A) SEM image of a whole SF-268 neurosphere (1000×). (B) SEM image of the neurosphere surface (2200×). (C–J), TEM images of inner and peripheral neurospheres. Details of thin membrane extroflection (C–D), interactions between electron-dense and electron-lucent cells (E), details of cell–cell adhesion (F), suffering sites in the inner spheres, with necrotic (G) and apoptotic (H) cells, living cells, and details of the mitochondrial apparatus in the inner spheres (I, J). Note the apoptotic cells at the periphery of a neurosphere (D) and the living cells close to the suffering sites (I). Bar: (C–E) and (G–I), 5 μm; (F and J), 1 μm.

**Fig 4:**
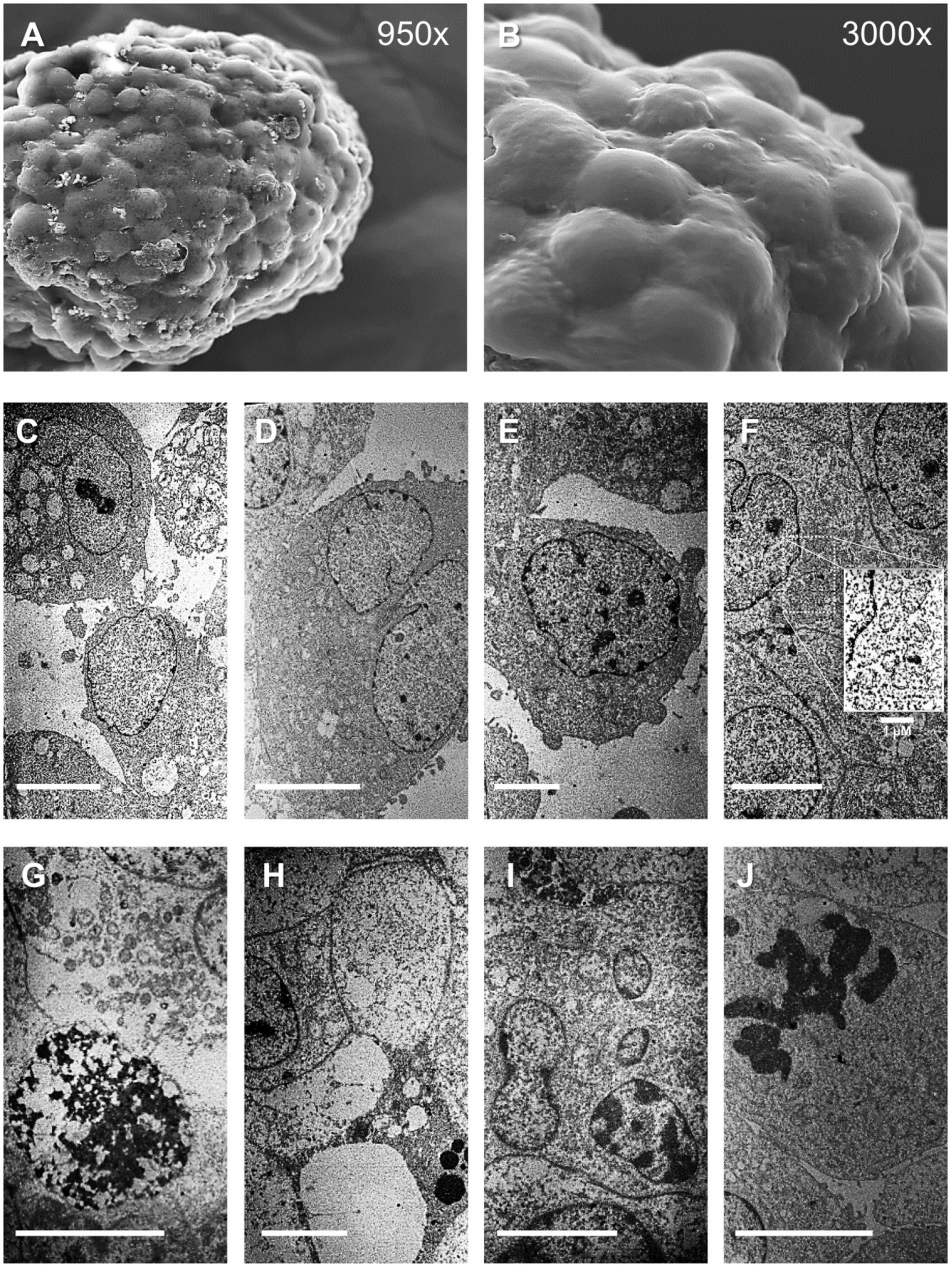
Representative images of NB t_8_ neurospheres. (A) SEM image of whole SK-N-SH neurosphere (950×). (B) SEM imaging of neurosphere surface (3000×). (C–J) TEM images of inner and peripheral neurosphere. Details of the cell vesicles, cell polarization, and detachment (C–E), living cells in the inner neurospheres and details of the mitochondrial apparatus (F), suffering sites in the inner spheres, with necrotic (G) and apoptotic cells (H and I), and an intrasphere mitotic event (J). Bar: (C–J), 5 μm.

Ultrastructural imaging with TEM and SEM showed broad cellular heterogeneity in the t_8_ neurospheres of both cell lines. SEM imaging of the neurosphere surfaces showed more packaged cells in the NB neurospheres than in the GBM neurospheres, whereas the peripheral cells of the GBM neurospheres had more thin membrane extroflections than the SK-N-SH neurospheres (Figs 3A, 3B, 4A and 4B). Adjacent electron-dense and electron-lucent cells and sporadic apoptotic cells were observed at the GBM neurosphere peripheries (Fig 3C–3E). The peripheral cells of the NB neurospheres were polarized, with more OsO_4_ staining in the cytoplasm than the inner cells, and contained dense granules, endocytic vesicles, and few membrane extroflections (Fig 4C–4E). Wide areas of cell death, characterized by membrane blebbing, cell wrinkling, apoptotic bodies, and wide nuclear compacted heterochromatin, were observed in the middle of the neurospheres of both cell lines (Figs 3G, 3H, 4G and 4H). However, active mitosis and living cells close to the inner stressed sites were also observed, in both the GBM and NB neurospheres (Figs 3G, 3I, 3J, 4F and 4J). Interestingly, these microenvironment-resistant cells in the hypoxic neurosphere core showed indented nuclei, large amounts of euchromatin and clearly visible mitochondrial apparatus.

### TMZ treatment and MTT assay of SRLP^+^ CSC-like and parental cell lines

**Fig 5:**
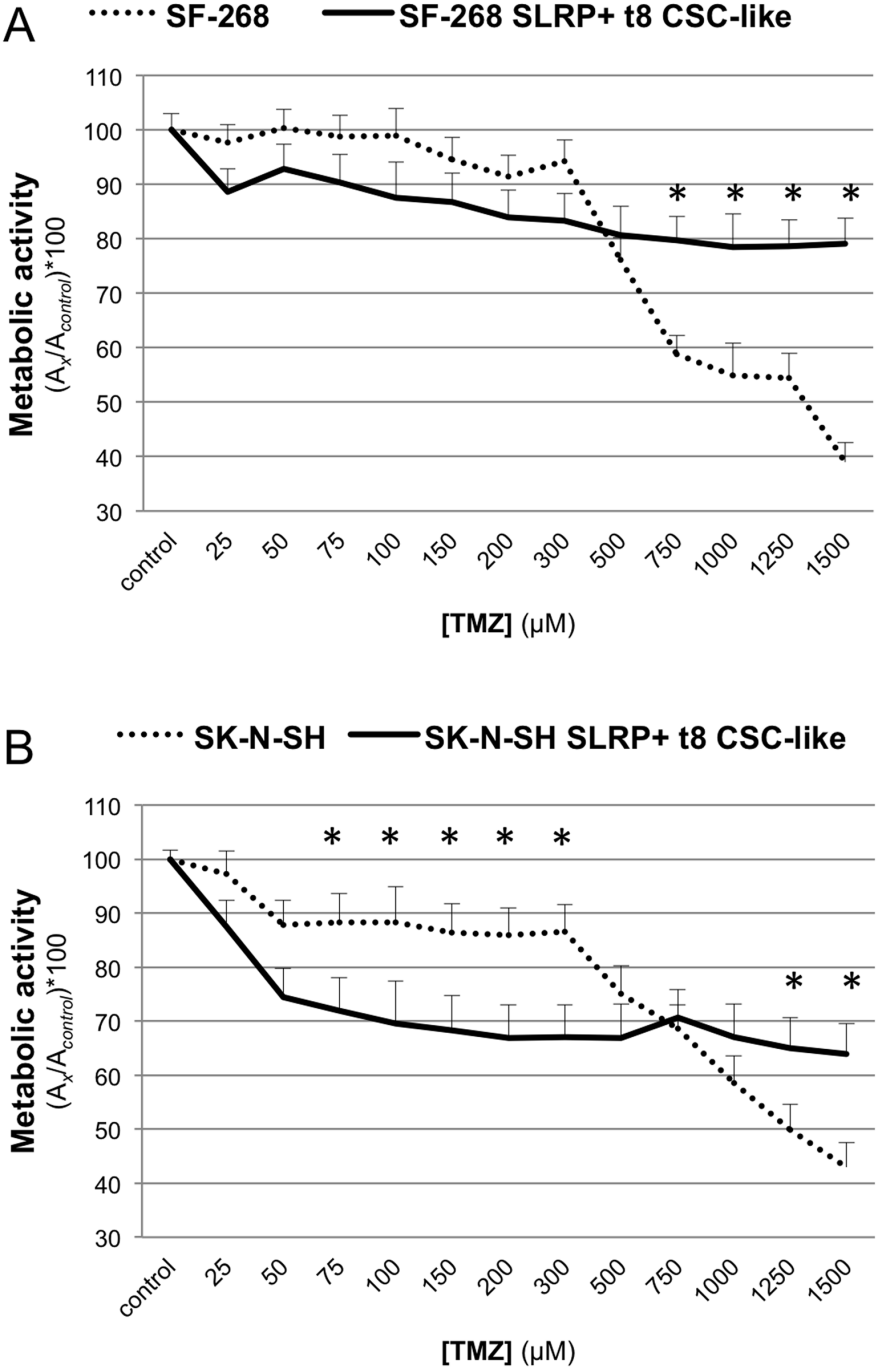
MTT assay after TMZ treatment. (A) SF-268 adherent cells *versus* SF-268 SLRP^+^ t_8_ CSC-like. (B) Adherent SK-N-SH cells *versus* SK-N-SH SLRP^+^ t_8_ CSC-like. Cell growth inhibition by low doses of TMZ (0–500 μM) was greater in the t_8_ CSCs-like than in the parental cell lines, with significance in SK-N-SH cells (*p < 0.05, Fig 5B). In contrast, cell growth inhibition by high doses of TMZ at, corresponding to pharmacological doses (> 750 μM), was greater in the parental cells than in the t_8_ CSCs-like cells in the CSC enrichments of both cell lines (*p < 0.05, Fig 5A and 5B). DMSO background was subtracted from samples and control values, and data shown as mean (SD) of [(A_samples_—A_DMSO_) / (A_control—_A_DMSO_)]*100 of four independent experiments.

Low-range TMZ concentrations (0–500 μM) did not induce significant differences in the metabolic activities of the SF-268 parental cells and SRLP^+^ t_8_ CSC-like. As shown in Fig 5A, the metabolic activity of the parental cells ranged from 76.14% to 100% (94.63% ± 7.10%), whereas metabolic activity of SRLP^+^ t_8_ CSC-like ranged from 80.71% to 100% (88.22% ± 5.43%). Higher concentrations of TMZ (750–1500 μM) induced significant differences between the SLRP^+^ t_8_ CSC-like and the parental cell line. At these concentrations, the metabolic activity of the SF-268 parental cell line ranged from 38.99% to 58.86% (51.80% ± 7.59%), whereas that of SF-268 SRLP^+^ t_8_ CSC-like ranged from 79.09% to 79.64% (78.94% ± 0.45%) (p<0.05). The SF-268 parental IC_50_ was around 1250 μM, and did not reach the IC_50_ of the SF-268 SRLP^+^ t_8_ CSC-like.

At low and high concentrations, TMZ induced significant differences in the metabolic activities of the SK-N-SH parental cells and the SRLP^+^ t_8_ CSC-like. As shown in Fig 5B, the metabolic activity of cells treated with 0–500 μM TMZ ranged from 75.08% to 100% (88.41% ± 6.70%) for parental cells and from 66.86% to 100% (74.70% ± 10.84%) for SRLP^+^ t_8_ CSC-like (p<0.05). However, in cells treated with 750–1500 μM TMZ, the metabolic activity switched and ranged from 42.91% to 68.56% (54.96% ± 9.61%) for parental cells and from 63.92% to 70.61% (66.64% ± 2.50%) for SRLP^+^ t_8_ CSC-like (p<0.05). The IC_50_ in SK-N-SH parental cells was around 1250 μM, and did not reach the IC_50_ of the SK-N-SH t_8_ SRLP^+^ CSC-like.

## Discussion

The stem cell theory of cancer is a new understanding of cancer development that considers oncogenesis to be aberrant organogenesis [56]. Because CSC-like are present in cancer as tumor-initiating cells and circulating tumor cells, they might interact with and react to the CSC niche, which may modulate the cell fates, contributing to cancer tissue heterogeneity and drug resistance [57]. This plasticity facilitates anchorage loss and cell motility, generating circulating tumor cells *via* the EMT in an instructive microenvironment. Different CSC subpopulations have been found within the same tumor [58], dispelling all doubt about the roles of CSCs in cancer heterogeneity and microenvironment modulation, and consequently in drug resistance [59, 60]. Several previous studies reported SLRP proteins in breast [61], pancreatic [62], colorectal [63], uterine cervical [64], prostate [30], and lung cancers [65], among others, highlighting the controversial roles of DCN and LUM in cancer biology. Our results provide the first evidence of the relevance of DCN and LUM to CSC biology, demonstrating significantly increased mRNA and protein levels of both SLRPs in GBM and NB CSC-like. However, while LUM mRNA and protein increased in t_8_ neurospheres, DCN mRNA increased at t_8_ and DCN protein at t_12_. This fact could be explained by differences between DCN and LUM in intrasphere trapping and maybe by CSC specific post-transcriptional regulation mechanisms which are still unknown. In addition, the influence of growth factors and their receptors, such those of EGF and TGF-β1, in DCN modulation and trafficking has been showed in adult cells [66, 67], suggesting putative crosstalk of DCN and growth factors pathways in CSC, which deserve further mechanistic investigations.

The 3D tumorsphere cultures in conditioned serum-free media, which constitute a methodological evolution of the neurosphere cultures used in neural stem and progenitor cell research [68–70], are considered representative *in vitro* cancer models useful in the CSC-like enrichment of primary [71–75] and established cancer cell lines [76, 77]. Here, this model was used to achieve neurosphere-based serum-free CSC enrichment of human GBM and NB cancer cell lines, enhancing cell plasticity through cell dedifferentiation towards a stem-like phenotype. Neurospheres of both cell lines showed heterogeneity in their cell size, ultrastructure, and proliferation, confirming the relevance of CSC-like in the maintenance of cell heterogeneity. Ultrastructural imaging of the neurospheres revealed both electron-dense and electron-lucent cells, which implies the presence of cells at different stages of differentiation, with peripheral cells enriched in endocytic vesicles, probably for vesicle-mediated internalization of DCN by RTK [66], and cell-specific signs of differentiation, particularly dense granules in NB cells and thin membrane extroflections in GBM cells. The presence of living inner-neurosphere cells after CSC enrichment for 8 days suggest that only a fraction of the inner cells evade apoptosis or necrosis in the hypoxic microenvironment of the neurosphere core. Interestingly, CSC-like with the highest levels of DCN and LUM mRNA, isolated from heterogenic t_8_ neurospheres, switched towards a quiescent phenotype in the soft-agar-grown secondary neurospheres, showing reduced cell proliferation and apoptosis. The SLRP^+^ CSC-like still partly grew as loose cell aggregates in the DMEM soft agar-grown secondary cultures, suggesting residual CSC-like evasion of cell death. Moreover, the SLRP^+^ CSC-like acquired TMZ resistance, as shown in an MTT-based assay. Despite the higher proliferation rate of the parental cell lines *vis-à-vis* the slow cycling of the CSC-like at low doses of TMZ, significant resistance to high-dose TMZ was observed in the SLRP^+^ CSC-like of both cell lines, which could be a microenvironment-related phenomenon and/or attributable to the overexpression of multidrug-resistance or DNA repair genes in the CSC-like [78, 79]. On the other hand, the lower cell viability of CSC-like than parental cells at low TMZ concentrations might be result of underestimation due to the function of the ABC transporters in pumping out of the cells the MTT molecules.

Interestingly, quiescent CSC-like seem to be involved in the EMT and the mesenchymal–epithelial transition (MET) and in cancer dormancy, which have been closely associated with drug resistance [80]. Our data, showing the anchorage-independent growth, resistance to high concentrations of TMZ, and lower cell proliferation of t_8_ CSC-like relative to those of the parental cell lines, suggest that GBM and NB cells can acquire a quiescent stem-like phenotype. These results indicate a putative relation between DCN and cell quiescence which has been observed in other cellular models [81], and that SLRPs members, in particular the LUM proteoglycan, could play a role in the CSC microenvironment and in cancer dormancy [82]. However, further studies of the pathophysiologic role of DCN and LUM in CSC will be necessary.

The tumor mass is mainly composed of differentiated tumor cells. In metastatic cancer, the MET program fosters epithelial-like cell proliferation of homed circulating tumor cells. Previous studies have shown that DCN inhibits glioma growth and cell differentiation. In contrast, DCN has been reported to play protective and antiapoptotic roles in glioma cell lines exposed to hypoxic microenvironments. Inherent to this study, the formation of 3D neurospheres necessarily generates a hypoxic microenvironment in the inner regions, which is why some cells inside the neurospheres undergo apoptosis/necrosis. It is well known that hypoxia plays a critical role in CSCs and niche maintenance, promoting hypoxia-inducible factor (HIF)-dependent reprogramming of the differentiated tumor cells towards a CSC-like phenotype [83]. Interestingly, HIF downstream effectors inhibit NB cell differentiation and were reported to co-localize with neural crest and stem cell markers in the perivascular niche in NB biopsy samples [84]. Here, we report the presence of both suffering and living cells in the hypoxic microenvironment of the inner neurosphere, and that the living inner neurosphere cells are resistant to TMZ. Hence, according to the studies abovementioned and the proof-of-concept of the known SLRP anticancer activity, DCN and LUM could play dual microenvironment-dependent roles in the maintenance of CSCs, inhibiting the growth of epithelial-like proliferative cells, but concomitantly promoting the survival and stem-like properties of residual CSC-like, including TMZ resistance, quiescence, and the maintenance of heterogenic cancer cell phenotypes. Therefore, our data support microenvironment-dependent protective roles for SLRPs in both GBM and NB CSC-like.

In addition to the known ECM remodeling and soluble factors (TGF-β, tumor necrosis factor α, FGF) and membrane receptors (RTKs, Toll-like receptors 2–4) binding activities, we propose a pivotal role for the SLRP proteoglycans in neurosphere generation, CSC niche regulation, and the maintenance of a quiescent stem-cell-like phenotype, and consequently in cell fate and drug resistance of CSC-like. The SLRP expression patterns in NB CSCs may also indicate that the developmental and oncogenic EMT programs are actively cross-linked.

Further functional and clinical studies should clarify the roles of SLRPs in CSC biology and in cancer maintenance. More accurate evaluations of the SLRPs in GBM and NB biopsy specimens in terms of the CSC niche are required to determine the clinical potential of SRLPs, which may inspire niche-targeted cancer therapies in the fight against undifferentiated SRLP^+^ malignant cancers.
